# Genome-Wide Identification of the CBL-CIPK Gene Family in the Ice Plant and Functional Analysis of Salt Stress Tolerance

**DOI:** 10.3390/life15091476

**Published:** 2025-09-19

**Authors:** Can Wang, Nan Li, Haifeng Sun, Tianyue Xu, Jinghua He, Chenhao Zhang, Zipeng Meng, Xinyao Zhang, Rong Zhou, Yingchao Zhang, Xiaoming Song

**Affiliations:** 1 School of Life Sciences, School of Basic Medical Sciences, Key Laboratory for Quality of Salt Alkali Resistant TCM of Hebei Administration of TCM, North China University of Science and Technology, Tangshan 063210, China; 15256450120@163.com (C.W.); limanxi1989@163.com (N.L.); xutianyue@stu.ncst.edu.cn (T.X.); 18332979055@163.com (J.H.); zchenhao1220@163.com (C.Z.); meng15856701279@163.com (Z.M.); 13612065377@163.com (X.Z.); 2 Haidu College, Qingdao Agricultural University, Qingdao 265200, China; sunhdxy@163.com; 3 College of Horticulture, Nanjing Agricultural University, Nanjing 210095, China; rong.zhou@food.au.dk; 4 Department of Food Science, Aarhus University, Agro Food Park 48, DK-8200 Aarhus, Denmark; 5 National Center of Technology Innovation for Comprehensive Utilization of Saline-Alkali Land, Dongying 257300, China

**Keywords:** ice plant (*Mesembryanthemum crystallinum*), CBL gene family, CIPK gene family, phylogeny, expression analysis, three-dimensional structure

## Abstract

**Background**: The ice plant (*Mesembryanthemum crystallinum* L.) is a typical halophyte with remarkable stress resistance traits, including salinity and alkalinity tolerance. As a crucial signaling transduction pathway for plant responses to environmental stress, the CBL-CIPK signaling system plays a key role in regulating plant stress resistance. **Methods**: This study systematically analyzed the composition characteristics of the CBL and CIPK gene families across 24 plant species, including the ice plant, using comparative genomics approaches. **Results**: A total of 297 CBL and 561 CIPK gene family members were identified across the 24 species. Within the ice plant genome, 9 CBL and 18 CIPK genes were identified. Compared to model plants like *Arabidopsis thaliana*, the ice plant possesses a relatively higher number of CIPK genes, which may be related to its specific adaptation to saline–alkaline environments. Phylogenetic analysis indicated that the ice plant CBL and CIPK genes could be classified into three and four subfamilies, respectively. Expression analysis revealed that several genes (e.g., *McCBL1*, *McCBL4*, *McCIPK1*, *McCIPK2*) were significantly upregulated under salt stress, suggesting their important roles in the salt stress response. Notably, ice plant CBL and CIPK genes exhibit significant structural diversity. For instance, *McCBL3* contains significantly more CDS regions than other members, while CIPK genes can be divided into two types: single-CDS type and multi-CDS type. This structural variation may be associated with functional divergence during the evolution of the gene family. Furthermore, three-dimensional (3D) structure prediction showed that CBL proteins primarily consist of EF-hand domains and α-helices, whereas CIPK proteins additionally contain β-sheet domains, implying that this structural difference may be related to their distinct regulatory mechanisms. **Conclusions**: This study provides an important theoretical basis for a deeper understanding of the molecular mechanisms underlying the CBL-CIPK signaling pathway in the saline–alkaline stress response of the ice plant.

## 1. Introduction

The ice plant (*Mesembryanthemum crystallinum* L.), as a halophyte of significant economic and ecological value, has garnered widespread attention in recent years [1,2]. Rich in various nutrients and medicinal components, the ice plant also possesses unique salt tolerance mechanisms, demonstrating important research value and application potential against the backdrop of increasing global soil salinization [3,4].

The ice plant is rich in various bioactive substances such as D-pinitol, retinol, and folic acid, with the content of some nutrients reaching 2–3 times that of common vegetables [5,6]. These compounds possess multiple physiological functions, including improving liver function and maintaining visual health. Globally, the ice plant has been developed as a functional food, pharmaceutical raw material, and cosmetic ingredient [7]. It has achieved large-scale cultivation in countries like Japan, becoming an economically valuable crop with significant development potential [8].

More importantly, in the context of increasingly severe global soil salinization, the ecological value of the ice plant as a typical halophyte is becoming increasingly prominent. Statistics indicate that global saline–alkaline land area has reached 954 million hectares [9]. This seriously restricts the sustainable development of agriculture. Due to its multiple salt tolerance mechanisms, the ice plant can complete its entire life cycle in high-salinity environments with concentrations exceeding 200 mmol/L. Firstly, its specialized salt bladders effectively sequester excess salt. Secondly, it maintains cellular homeostasis by regulating ion balance and accumulating osmoprotectants such as proline and betaine. Additionally, the ice plant possesses an efficient antioxidant system and flexible photosynthetic pathway switching capability [2,3,10]. Under favorable conditions, the ice plant performs C3 photosynthesis, but under stresses like salt stress, its carbon assimilation pathway can switch from C3 to CAM (Crassulacean Acid Metabolism) [11], thereby improving water use efficiency and stress resistance. These characteristics enable it to adapt to high-salt environments and contribute to absorbing soil salts for saline–alkaline soil reclamation.

Plants encounter a variety of environmental stresses during their growth and development, which can induce the expression of multiple responsive genes [12,13,14]. Plants respond to environmental stresses through complex signal transduction networks. And calcium ions (Ca^2+^) act as key secondary messengers, which play a central role in regulating stress responses [15,16]. Plant cells contain three main types of Ca^2+^ sensors: Calmodulin (CaM), Calcineurin B-like proteins (CBL), and Calcium-Dependent Protein Kinases (CDPKs) [17,18]. Among these, the signaling pathway composed of CBL and CBL-interacting protein kinases (CIPK) holds a special position in plant stress responses [19,20,21]. CBL proteins specifically bind Ca^2+^ via their three EF-hand domains, with N-terminal myristoylation and palmitoylation modifications determining their subcellular localization [22]. CIPK interacts with CBL via its C-terminal NAF/FISL domain, which also maintains precise regulation of kinase activity through an autoinhibition mechanism [23]. The CBL-CIPK complex participates in regulating various stress resistance-related physiological processes, such as the SOS pathway, ABA signaling transduction, and ROS metabolism, by phosphorylating downstream target proteins [24,25,26].

Halophytes are considered ideal model systems for studying plant salt tolerance mechanisms due to their unique salt response and tolerance traits [26,27]. As a typical halophyte with significant saline–alkaline tolerance, the ice plant exhibits important value in saline–alkaline agriculture development. Although its whole-genome sequencing has been completed, the molecular mechanisms of the key salt tolerance regulatory system, the CBL-CIPK signaling pathway, remain unclear. This study systematically identified members of the CBL and CIPK gene families in the ice plant, combined with domain analysis, chromosomal localization, gene structure analysis, phylogenetic analysis, and transcriptomic data integration, to comprehensively dissect the regulatory network of this signaling pathway in salt tolerance response. This lays a theoretical foundation for elucidating the molecular mechanisms by which the CBL-CIPK signaling pathway mediates saline–alkaline stress tolerance in the ice plant, thereby contributing to understanding the adaptive mechanisms of plants like the ice plant to stresses like salt stress and providing a theoretical basis for breeding new saline–alkaline-tolerant crop varieties and improving plant stress responses.

## 2. Materials and Methods

### 2.1. Identification and Phylogenetic Analysis of CBL-CIPK Genes

Using TBtools (TBtools_windows-x64_1_0987663) software [28], protein files from 24 species, including the ice plant, were compared against *Arabidopsis thaliana* Calcineurin B-like proteins (CBLs) obtained from the TAIR database. Comparisons were made with the reported number of CBL genes in Chinese cabbage (*Brassica rapa*), grape (*Vitis vinifera*), pineapple (*Ananas comosus*), and rice (*Oryza sativa*) to determine BLASTP screening parameters: Outfmt: table; NumofThreads: 2; E-value: 1 × 10^−10^; NumofHits: 500; NumofAligns: 250; these values refer to the previous studies [29,30]. The initially identified protein sequences were validated using the Pfam, SMART, and CDD databases [31].

The HMMER model was used for the initial identification of plant CIPK gene family members, referring to the previous studies [32,33], retaining proteins containing both the Pkinase (Pfam NO. PF00069) and NAF (Pfam NO. PF03822) domains.

OrthoFinder (V 2.0) software under Linux was used to analyze the 24 species and construct a phylogenetic tree [34]. Protein sequence files of the CBL and CIPK gene families from the analyzed species were processed using MAFFT software (v7.526) [35]. Subsequently, FastTree (V 1.6.0) software was used to process and analyze the MAFFT-aligned protein files and construct the tree [36]. The obtained tree files were visualized using the online website iTOL (Interactive Tree of Life) for pruning, annotation, description, and more detailed subfamily classification [37].

### 2.2. Analysis of Physicochemical Properties of CBL-CIPK Proteins

The physicochemical properties of all identified CBL and CIPK proteins in the ice plant were predicted using TBtools software (Protein Parameter Calc module) [28]. Additionally, the WoLF PSORT online tool (https://wolfpsort.hgc.jp/ (accessed on 23 December 2024)) was used to predict the subcellular localization information for all identified CBL and CIPK genes.

### 2.3. Analysis of Conserved Domains, Motifs, and Gene Structure of CBL-CIPK Gene Family Members

Using MEGA11.0 (https://www.megasoftware.net/ (accessed on 9 September 2024)) software, a phylogenetic tree of ice plant CBL and CIPK gene family members was constructed using the Neighbor-Joining (NJ) method [38]. The Newick file was saved and visualized using the online website iTOL (https://itol.embl.de/ (accessed on 5 May 2025)) [37]. Conserved motif analysis was performed using the online website MEME (https://meme-suite.org/meme/tools/meme (accessed on 9 August 2024)) [39]. Gene structure (exon–intron) analysis was performed using the TBtools software (Visualize Gene Structure (from GTF/GFF3 File) module) [28].

### 2.4. Chromosomal Distribution and Intraspecies Collinearity Analysis of Ice Plant CBL and CIPK Genes

Using TBtools software (Gene Location plugin) [28], based on the ice plant genome annotation information, all identified CBL and CIPK genes were mapped to chromosomes, and the collinear relationships of CBL and CIPK genes within the species were constructed and visualized.

### 2.5. Analysis of Cis-Acting Elements in Ice Plant CBL and CIPK Genes

The online website PlantCARE was used to extract promoter sequences 2000 bp upstream of the start codon of ice plant CBL and CIPK genes for cis-acting element prediction [40]. The predicted cis-regulatory elements were then visualized using TBtools software [28].

### 2.6. Construction of 3D Models for Ice Plant CBL-CIPK Proteins

AlphaFold3 (https://alphafoldserver.com/ (accessed on 18 January 2025)) was used to construct 3D models of ice plant CBL and CIPK proteins (entity type: protein; copies: 1).

### 2.7. Protein–Protein Interaction Network Analysis

The online STRING database (https://cn.string-db.org (accessed on 11 January 2025)) was used, based on known *A. thaliana* homologs, to predict the protein interaction network and gene co-expression relationships of ice plant CBL and CIPK proteins. Cytoscape (V3.10.2) was used to visualize the resulting network [41].

### 2.8. Gene Expression Profile Analysis

Ice plant transcriptome data was downloaded from previous reports [2,10]. The expression heatmap of ice plant CBL and CIPK genes in different tissues and under salt stress was drawn using TBtools software (HeatMap module) [28].

## 3. Results

### 3.1. Identification and Phylogenetic Analysis of CBL-CIPK Gene Family Members in 24 Plant Species

**Figure 1:**
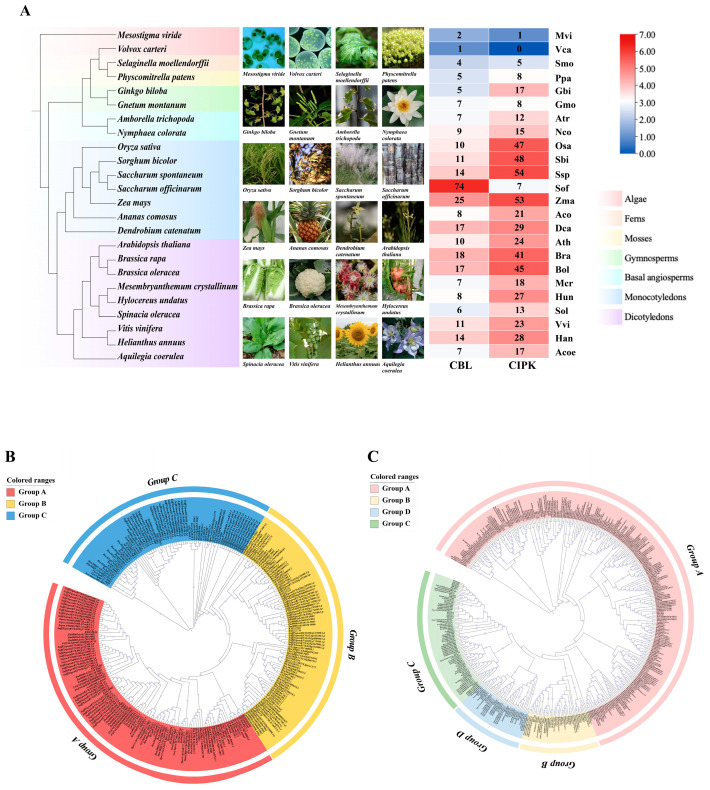
Evolutionary analysis of the plant CBL-CIPK gene family. (**A**) Genome-wide identification heatmap of CBL and CIPK gene family members in 24 plant species. (**B**) Phylogenetic tree of CBL genes. (**C**) Phylogenetic tree of CIPK genes.

This study selected 24 representative plant species for systematic identification and evolutionary analysis of the CBL and CIPK gene families. Phylogenetic analysis results (Figure 1) showed that the number of CBL-CIPK gene family members exhibited a clear increasing trend with the evolutionary advancement of plants. Notably, the number of CBL gene family members was generally lower than that of CIPK, but sugarcane (*Saccharum officinarum*) showed a significant exception, with its CBL family reaching 74 members, while only 7 CIPK genes were identified.

Gene number heatmap analysis further revealed the following: Algae and ferns overall showed low numbers of genes (blue), with fewer members in both CBL and CIPK gene families. Starting from gymnosperms, the number of CBL-CIPK gene family members significantly increased. Monocots reached the peak number of gene family members. In contrast, dicots generally had fewer CBL-CIPK gene family members than monocots. These results systematically reveal the expansion pattern of the CBL-CIPK gene family during plant evolution.

Phylogenetic analysis results (Figure 1B) showed that CBL genes from different species clustered with different *A. thaliana* subfamily members into three stable evolutionary branches, allowing them to be classified into three subfamilies: Group A (red), Group B (yellow), and Group C (blue). From the overall structure of the tree, the branches of different subfamilies diverged from a common ancestral node, indicating that the CBL gene family underwent multiple divergence events during evolution. Branches within each subfamily were further subdivided, reflecting continuous variation and differentiation of genes during evolution, forming multiple related small branches. The CIPK gene family (Figure 1C) could be divided into four subfamilies: Group A (pink), Group B (yellow), Group C (green) and Group D (blue). Group A accounts for the largest proportion in the phylogenetic tree. The number of gene members of Group A in this subfamily is relatively large. They share a close evolutionary relationship. They were likely formed through a series of variations and differentiations from a common ancestral gene. There may also be some commonality in their functions.

Statistical results of plant CBL-CIPK gene family members (Table 1) indicated the following:

C3 plants analyzed in this study included the monocot rice (*Oryza sativa*) and several dicot species: Arabidopsis thaliana, Chinese cabbage (*Brassica rapa*), cabbage (*Brassica oleracea*), spinach (*Spinacia oleracea*), grape (*Vitis vinifera*), and sunflower (*Helianthus annuus*). The number of CBL genes in these species fluctuated between 10 and 18, with rice (*Oryza sativa*) and *Arabidopsis thaliana* containing 10 genes, and Chinese cabbage (*Brassica rapa*) possessing 18. These differences in CBL family size are likely attributed to variations in genomic characteristics among species. The CIPK gene family size in C3 plants exhibited significant variation (23–45 genes), which is attributed to divergent evolutionary expansion driven by species-specific factors and environmental pressures.

C4 Plants: C4 plants analyzed in this study included the monocot species sorghum (*Sorghum bicolor*), wild sugarcane (*Saccharum spontaneum*), sugarcane (*Saccharum officinarum*), and maize (*Zea mays*). The number of CBL genes in these species ranged from 11 to 74. In particular, sugarcane (*Saccharum officinarum*) contained 74 CBL genes, a markedly higher count likely attributable to its large genome size and the presence of repetitive sequences that facilitated extensive gene duplication. In contrast, sorghum (*Sorghum bicolor*) possessed only 11 CBL genes. The number of CIPK genes exhibited a more constrained range and varied between 47 and 54 across these species. This relative concentration in CIPK gene numbers suggests that, during the evolution of the C4 photosynthetic pathway, the CIPK gene family may have undergone a comparatively conservative expansion to meet the requirements of efficient photosynthetic metabolism.

CAM Plants: CAM plants analyzed in this study encompassed both monocots and dicots, including pineapple (*Ananas comosus*), orchid (*Dendrobium catenatum*), ice plant (*Mesembryanthemum crystallinum*), pitahaya (*Hylocereus undatus*), and Colorado blue columbine (*Aquilegia coerulea*). The number of CBL genes in these species ranged from 7 to 17 and exhibited considerable variation. This wide span reflects the diverse evolutionary history of CAM plants and their distinct adaptations to specialized habitats, such as drought environments. In comparison, the number of CIPK genes ranged between 17 and 29 across the examined species and showed a more constrained variation.

Overall comparison: Within the CBL gene family, sugarcane in C4 plants, due to unique genomic features, has a far higher CBL count than C3 and CAM plants; C3 plants show relatively dispersed CBL numbers; CAM plants have a large span in CBL numbers. The evolution of the CBL gene family in different photosynthetic types is driven by their own genomic background and ecological niches. In the CIPK gene family, C4 plants have relatively concentrated and overall higher CIPK numbers, potentially related to the complex enzymatic reactions and metabolic coordination requirements of the C4 photosynthetic pathway; C3 plants have a wide range of CIPK numbers, reflecting gene family differentiation under diverse survival strategies; CAM plant CIPK numbers are adapted to their specific photosynthetic rhythm, with facultative CAM plants exhibiting both flexibility and specificity in their CIPK gene family. Plants of different photosynthetic types adjust the number of CBL and CIPK gene members to adapt to their respective photosynthetic physiological needs, with gene family evolution deeply linked to photosynthetic strategies and ecological adaptation.

To deeply investigate the role of the plant CBL-CIPK gene family in saline–alkaline tolerance, this study focused on the typical halophyte ice plant as the main research subject for further detailed analysis of its CBL-CIPK gene family members.

### 3.2. Chromosomal Localization of Ice Plant CBL-CIPK Gene Family Members

**Figure 2:**
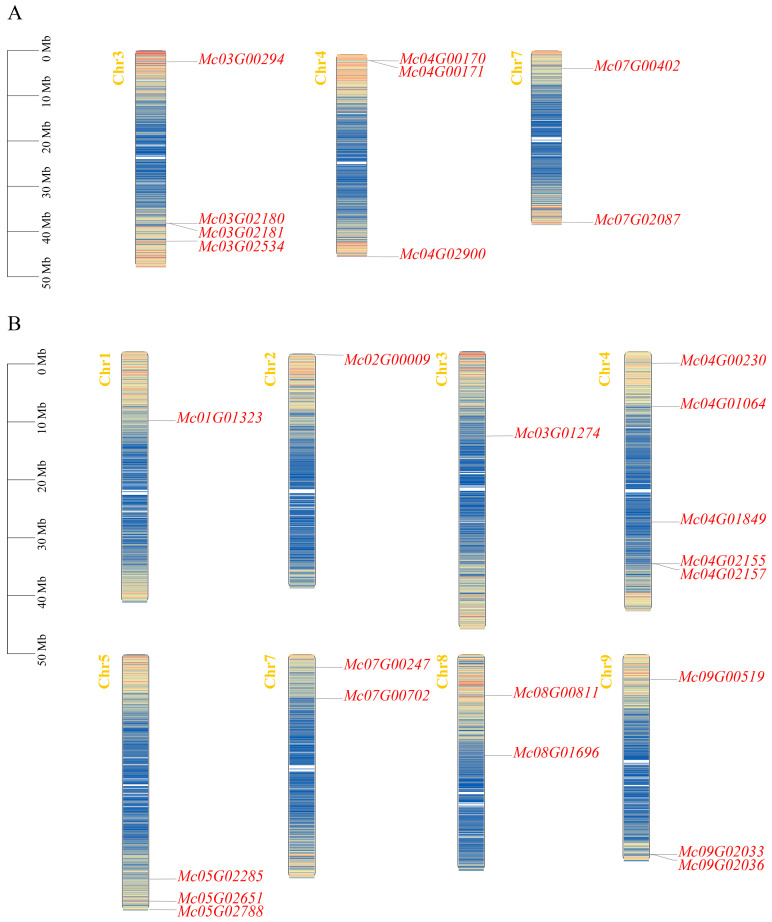
Chromosomal localization of ice plant CBL-CIPK genes. (**A**) Chromosomal localization of ice plant CBL gene family members. (**B**) Chromosomal localization of ice plant CIPK gene family members.

A total of 27 CBL-CIPK gene family members were identified in the ice plant genome, comprising 9 CBL and 18 CIPK genes. Based on their chromosomal distribution positions (Figure 2), they were named *McCBL1* to *McCBL9* and *McCIPK1* to *McCIPK18*. *McCBL* genes are distributed on chromosomes 3, 4, and 7 of the ice plant. *McCIPKs* are distributed on chromosomes 1, 2, 3, 4, 5, 7, 8, and 9. Chromosomal localization results show that on chr3 (chromosome 3), the three genes *McCBL2*, *McCBL3*, and *McCBL4* are located at positions approximately 30 MB to 50 MB, forming a distinct gene cluster, suggesting these genes may have arisen through tandem duplication.

### 3.3. Analysis of Subfamilies, Conserved Motifs, and Gene Structure of Ice Plant CBL-CIPK Genes

**Figure 3:**
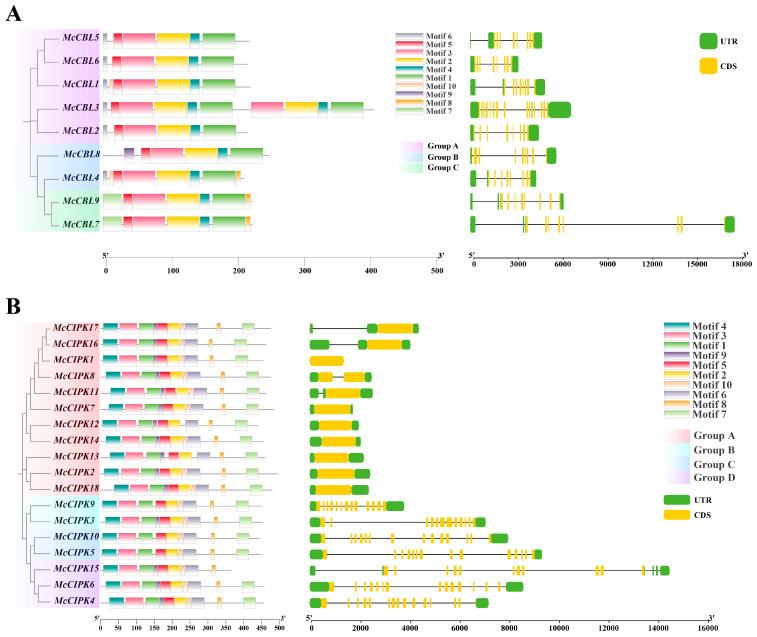
Analysis of conserved motifs and gene structure of ice plant CBL-CIPK genes. (**A**) CBL gene family. (**B**) CIPK gene family.

Phylogenetic analysis based on the Maximum Likelihood (ML) method (Figure 3) showed that the nine CBL genes of the ice plant could be divided into three distinct evolutionary subgroups. Gene structure analysis indicated that family members have relatively conserved exon–intron structures, with the number of coding sequences (CDS) ranging between 8 and 15. Notably, *McCBL3* has a significantly higher number of CDS regions than other members.

The phylogenetic tree of the 18 ice plant CIPK genes, constructed based on the Maximum Likelihood (ML) method (Figure 3), could be divided into four distinct evolutionary subgroups (Group I, II, III, IV). Gene structure analysis showed that ice plant CIPK genes have relatively conserved exon–intron structure but exhibit significant variation in CDS number (1–14). Unlike the CBL gene family, CIPK genes can be clearly divided into two categories: one containing only a single CDS (11 members), and the other being CDS-rich (7 members), with CDS numbers ranging from 11 to 14. This structural feature suggests that the CIPK gene family may have undergone significant functional divergence during evolution.

### 3.4. Physicochemical Properties and Subcellular Localization of Ice Plant CBL-CIPK Proteins

CBL protein length varied within a small range (Table 2). Except for *McCBL3* (410 amino acids, predicted molecular weight 47.3 kDa), the other eight members ranged from 213 to 252 amino acids, with molecular weights of 24.5–28.7 kDa. Theoretical isoelectric points (pI) ranged from 4.64 to 5.24, all being acidic (pI < 7). Among the nine proteins, four members had instability indices > 40, indicating lower structural stability; *McCBL6* had an instability index of 29.47, indicating higher stability. The aliphatic amino acid index ranged from 84.18 to 98.33, with *McCBL8* being the highest (98.33), suggesting potentially better thermostability. The grand average of hydropathicity (GRAVY) values for all proteins were negative, confirming their hydrophilic nature [42]. Subcellular localization prediction indicated that most are localized to the cytoplasm, while *McCBL1* is mainly distributed in the nucleus, and *McCBL4* and *McCBL8* are primarily localized in the chloroplast.

CIPK protein length varied considerably (365–495 amino acids), with molecular weights of 41.3–55.3 kDa. Most (14) had pI > 7, being basic proteins; *McCIPK2*, *McCIPK5*, *McCIPK9*, and *McCIPK15* had pI < 7, being acidic proteins. Among the 18 proteins, 5 had instability indices > 40, indicating poorer stability; the remaining 13 had indices < 40, being relatively stable, with *McCIPK14* having the lowest (27.89). The aliphatic index ranged from 80.08 to 95.92 (*McCIPK3* highest, 95.92). GRAVY values for all proteins were negative, indicating hydrophilic proteins. Subcellular localization results showed eight in the cytoplasm, seven in the chloroplast, two on the plasma membrane, and one in the mitochondrion.

### 3.5. Analysis of Cis-Acting Regulatory Elements in Ice Plant CBL-CIPK Gene Family Members

**Figure 4:**
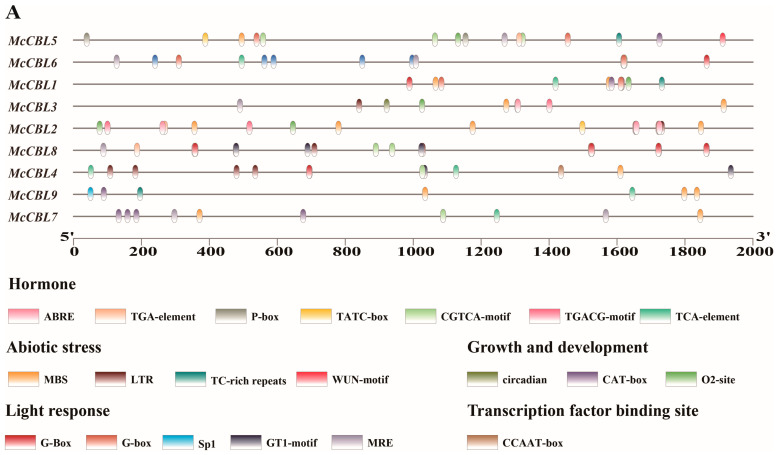

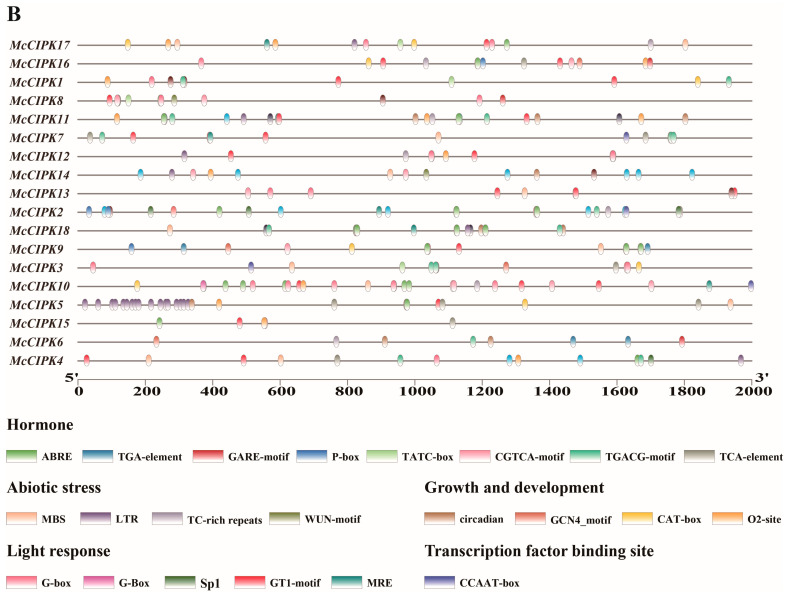
Analysis of cis-acting elements in promoters of ice plant CBL-CIPK gene family members. (**A**) CBL gene family. (**B**) CIPK gene family.

Bioinformatic prediction and analysis of cis-acting elements were performed on the promoter regions (2000 bp upstream of the start codon) of the CBL-CIPK genes in the ice plant (Figure 4).

*McCBL* promoters (Figure 4A) contain various functional elements and mainly include (1) phytohormone response elements (abscisic acid-responsive element (ABRE), auxin-responsive element (TGA-element), gibberellin-responsive element (P-box/TATC-box), methyl jasmonate-responsive element (CGTCA-motif/TGACG-motif) and salicylic acid-responsive element (TCA-element)); (2) abiotic stress response elements (drought-inducible element (MBS), low-temperature-responsive element (LTR), stress-responsive element (TC-rich repeat) and wound-responsive element (WUN-motif)); (3) growth- and development-related elements (circadian control element (circadian), Meristem expression element (CAT-box) and Zein metabolism regulation element (O2-site)); (4) light-responsive elements (G-box, Sp1, GT1-motif, MRE); and (5) transcription factor binding sites (MYB recognition site/CCAAT-box). Among these, methyl jasmonate-responsive elements (22), abscisic acid-responsive elements (ABRE, 21), and drought-inducible elements (MBS, 13) were the most abundant.

*McCIPK* promoters (Figure 4B) were also enriched with various cis-acting elements, classified similarly to *McCBLs*: (1) phytohormone-response elements; (2) abiotic stress response elements; (3) growth- and development-related elements (including the endosperm expression element GCN4_motif); (4) light-responsive elements; and (5) transcription factor binding sites (CCAAT-box). Among these, methyl jasmonate-responsive elements (total 62), abscisic acid-responsive elements (ABRE, 42), low-temperature-responsive elements (LTR, 23), and light-responsive elements (G-box, 38; GT1-motif, 25) were the most abundantly distributed.

### 3.6. Intraspecies Collinearity Analysis of Ice Plant CBL-CIPK Gene Family Members

**Figure 5:**
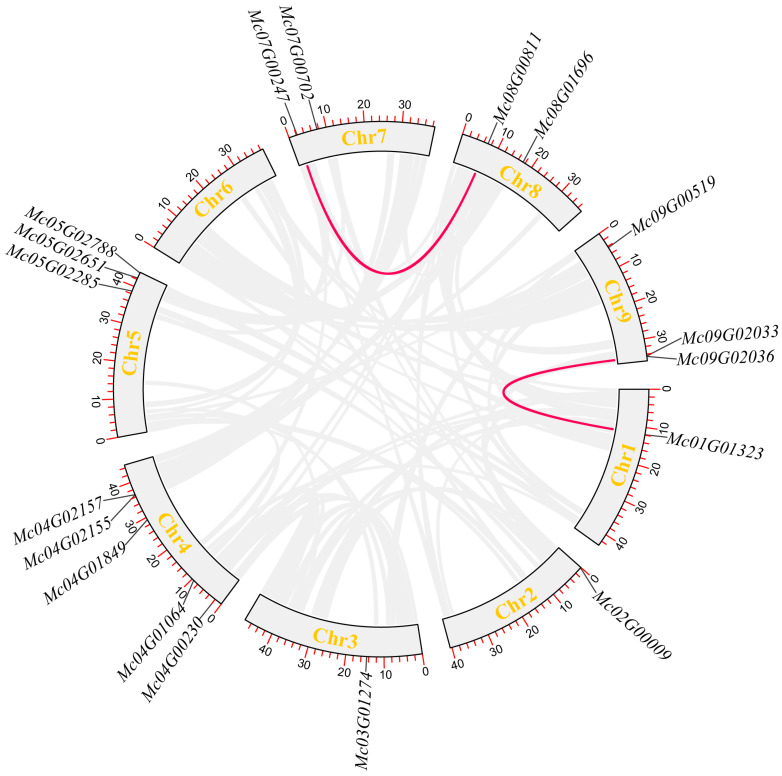
Intraspecies collinearity analysis of ice plant CIPK gene family.

Collinearity analysis was performed on ice plant CBL-CIPK gene family members (Figure 5). Results showed no significant collinear relationships among ice plant CBL family members, suggesting low homology between them. Homology among ice plant CIPK gene family members was also low, with collinearity detected only between *McCIPK1* and *McCIPK17*, and *McCIPK18*, and between *McCIPK12* and *McCIPK14*. This indicates that these collinear genes may be homologous and perform the same or similar functions in the saline–alkaline tolerance physiology of the ice plant.

### 3.7. Expression Profile Analysis of Ice Plant CBL-CIPK Genes

To study the expression of ice plant CBL-CIPK genes in roots, stems, and leaves, and under abiotic stress, this study analyzed the expression profiles of CBL-CIPK genes in different tissues of ice plants and their expression patterns under different salt concentration treatments based on transcriptome data.

**Figure 6:**
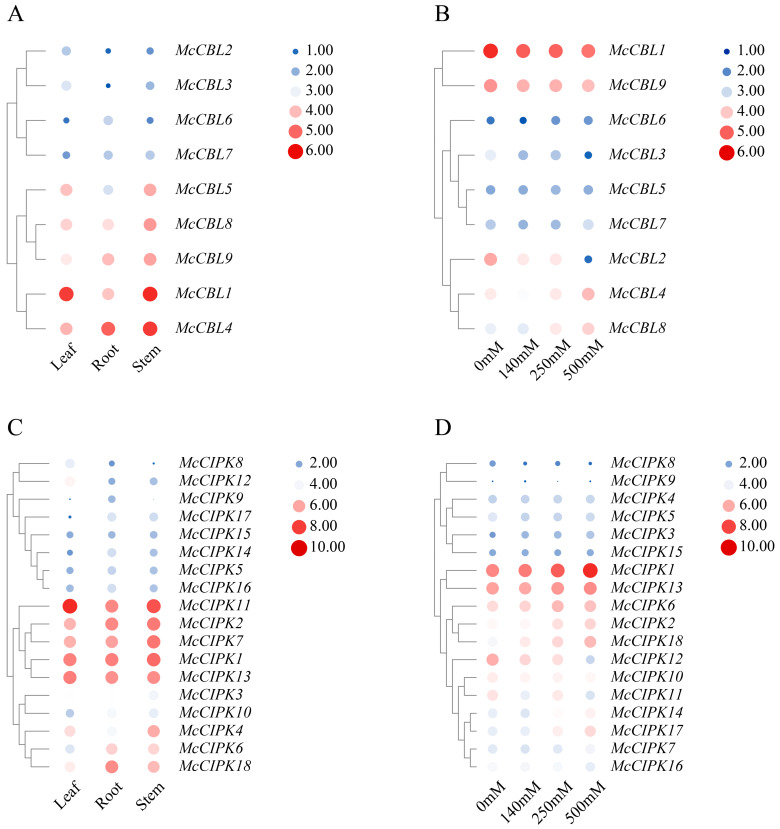
Heatmap of ice plant CBL-CIPK gene expression profiles. (**A**) CBL gene expression profile in different tissues. (**B**) Expression profile of CBL genes under salt stress. (**C**) CIPK gene expression profile in different tissues. (**D**) Expression profile of CIPK genes under salt stress.

Figure 6 shows that *McCBL1*, *McCBL4*, *McCBL8*, and *McCBL9* generally showed high expression across the tested tissues (overall red in the heatmap), with *McCBL1* and *McCBL4* exhibiting significant expression levels (dark red signal) in all three tissues: root, stem, and leaf. *McCBL5* showed a tissue-specific expression pattern, with higher expression in leaf and stem tissues (red signal) and relatively lower expression in root tissue (blue signal). In contrast, *McCBL2*, *McCBL3*, *McCBL6*, and *McCBL7* showed low expression (blue or light blue signal) in all tested tissues. These results indicate significant differences in expression among ice plant CBL gene family members across different tissues, possibly related to their functional differentiation.

Ice plant CIPK gene family members exhibited differential expression patterns across tissues. Among them, five genes (*McCIPK1*, *McCIPK2*, *McCIPK7*, *McCIPK11*, and *McCIPK13*) showed significantly high expression (red signal in heatmap) in root, stem, and leaf tissues. Notably, *McCIPK11* showed particularly strong expression signals (dark red) in leaf and stem tissues, suggesting its potential important role in these tissues. In contrast, eight genes including *McCIPK3*, *McCIPK4*, and *McCIPK8* generally showed low expression (blue or light blue signal) in all three tissues. This significant difference in expression patterns may reflect functional differentiation of CIPK gene family members in different tissues of ice plants.

Transcriptome data analysis based on different salt concentration treatments showed differential expression patterns among ice plant CBL gene family members. Heatmap analysis results (Figure 6) showed that *McCBL1* and *McCBL9* exhibited significantly high expression (red signal) under multiple salt concentration treatments. Notably, *McCBL1* showed a more intense expression signal (dark red) under specific salt treatments, indicating its expression level is significantly regulated by the degree of salt stress. *McCBL9* also showed relatively prominent expression patterns under some salt treatments. These expression features suggest that *McCBL1* and *McCBL9* may play key regulatory roles in the ice plant salt stress response. Notably, the expression levels of *McCBL4* and *McCBL8* showed a clear salt concentration-dependent upregulation trend. As the salt treatment concentration increased, the expression levels of these two genes gradually increased, indicating their potential involvement in the adaptive regulation process of ice plant to high-salt environments. This dose-dependent expression pattern further suggests that *McCBL4* and *McCBL8* may play important roles in the plant salt stress response pathway. In contrast, genes like *McCBL2*, *McCBL3*, and *McCBL7* generally showed low expression (blue or light blue signal) under various salt concentrations, suggesting these genes may not be directly involved in the salt stress response process. This differential expression pattern reflects functional divergence among CBL gene family members in plant salt stress adaptation.

Ice plant CIPK gene family members also showed differential expression patterns. Heatmap analysis results (Figure 6) indicate that the expression levels of seven genes, including *McCIPK1*, *McCIPK2*, *McCIPK6*, and *McCIPK13*, were positively correlated with salt treatment concentration, with their expression significantly upregulated as salt concentration increased. Notably, *McCIPK1* exhibited particularly significant induced expression under high-salt-stress conditions of 250 mM and 500 mM.

### 3.8. Construction of 3D Models and Protein Interaction Network Analysis for Ice Plant CBL-CIPK Proteins

**Figure 7:**
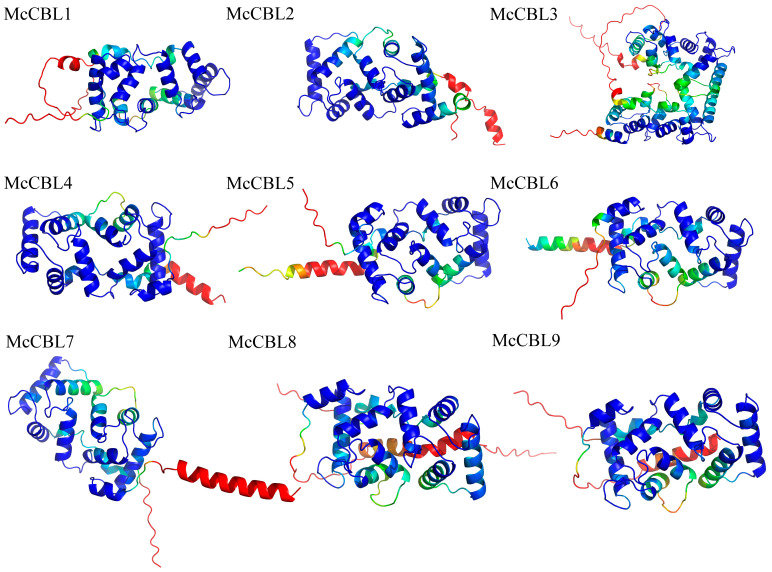
Three-dimensional models of ice plant CBL proteins. (Color represents prediction confidence: blue: very high (plDDT > 90); green: confident (90 > plDDT >70); yellow: low (70 > plDDT > 50); red: very low (plDDT < 50)).

**Figure 8:**
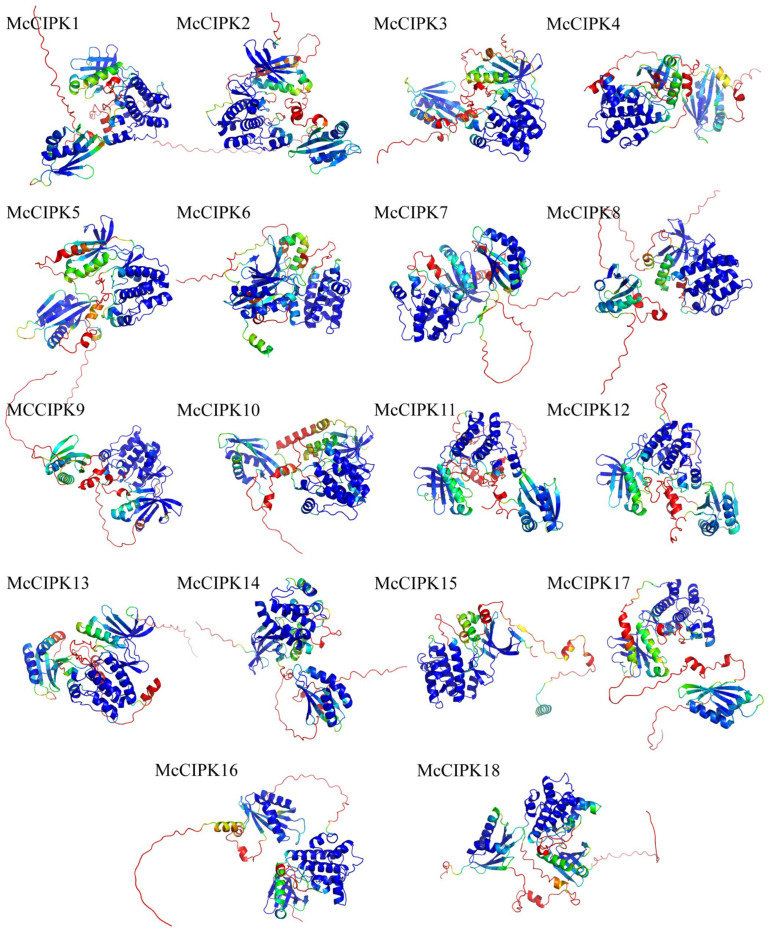
Three-dimensional models of ice plant CIPK proteins (color represents prediction confidence as in Figure 7).

AlphaFold3 was used to predict 3D models for ice plant CBL-CIPK proteins. Model quality assessment showed that the predicted pTM (predicted TM-score) values were all greater than 0.5, indicating high model reliability. Analysis results (Figure 7) showed that McCBL proteins all contain three or more EF-hand domains, and their three-dimensional structures are primarily composed of multiple α-helices. Compared to ice plant CBL proteins (Figure 8), McCIPK protein 3D structures all contain two additional β-sheet domains besides typical α-helices.

**Figure 9:**
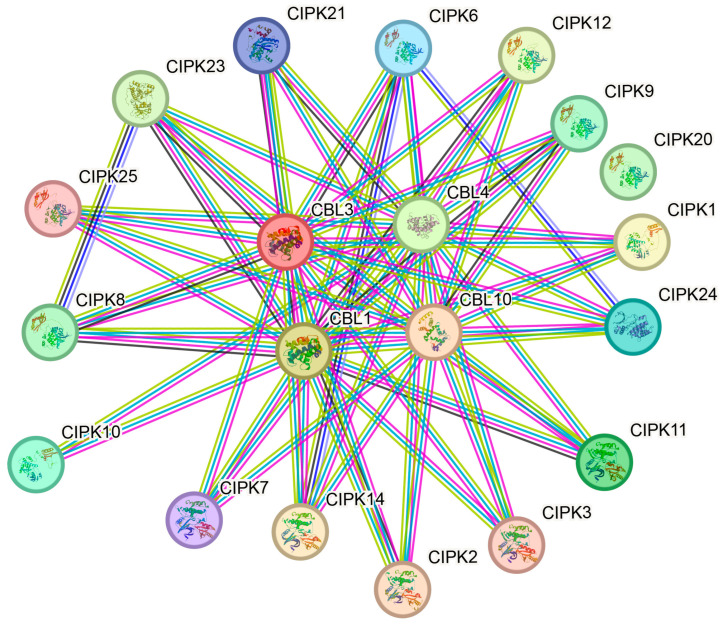
Predicted protein–protein interaction network of ice plant McCBLs and McCIPKs based on *Arabidopsis* homologs.

To explore the potential regulatory network of McCBL and McCIPK, this study constructed an McCBL-McCIPK protein interaction network based on *Arabidopsis* homologous genes. Analysis results (Figure 9) predicted significant functional associations between McCBL and McCIPK proteins, together forming a complex regulatory network system.

## 4. Discussion

The CBL-CIPK signaling pathway likely plays a key role in responding to abiotic stresses, particularly salt stress [43,44]. This study identified 9 CBL and 18 CIPK genes in the ice plant, a typical halophyte with remarkable saline–alkaline tolerance. Compared to model plants like *A. thaliana*, the ice plant possesses a relatively higher number of CIPK genes, which may be related to its specific adaptation to saline–alkaline environments. Phylogenetic analysis indicated that ice plant CBL and CIPK genes can be classified into three and four subfamilies, respectively. Some members (e.g., *McCBL1*, *McCBL4*, *McCIPK1*, *McCIPK2*) were significantly upregulated under salt stress, suggesting these genes may play core roles in the salt stress response. Notably, ice plant CBL and CIPK genes exhibit significant structural diversity. For instance, *McCBL3* has significantly more CDS regions than other members, while CIPK genes can be divided into single-CDS and multi-CDS types. This may reflect the functional differentiation of the gene family during the evolutionary process. Furthermore, 3D structure prediction showed that CBL proteins primarily consist of EF-hand domains and α-helices, but CIPK proteins additionally contain β-sheet domains.

Expression profile analysis revealed that the expression patterns of ice plant CBL and CIPK genes exhibit tissue specificity and salt concentration dependency. For example, genes like *McCBL1*, *McCBL4*, and *McCBL9* and *McCIPK1*, *McCIPK2*, and *McCIPK6* were significantly upregulated under salt stress, and the expression levels of some genes (e.g., *McCIPK1*) were positively correlated with salt concentration, suggesting their potential involvement in signal transduction under high-salt conditions. The expression of *McCBL4* and *McCBL8* gradually increased with rising salt concentration, indicating their potential roles in ion homeostasis regulation or osmo-protection. Some genes (e.g., *McCBL2*, *McCBL3*, *McCIPK3*) showed low expression under salt stress, possibly primarily involved in other physiological processes rather than the salt stress response.

Furthermore, promoter analysis found that the regulatory regions of ice plant CBL and CIPK genes are enriched with abscisic acid (ABRE), jasmonate (CGTCA-motif), and abiotic stress response elements (e.g., MBS, LTR), further supporting the regulatory role of these genes in stress responses. Collinearity analysis showed a lack of significant collinear relationships among ice plant CBL gene family members, while only a few CIPK gene members (e.g., McCIPK1/17/18, McCIPK12/14) exhibited collinearity, suggesting this gene family may have undergone complex gene duplication and functional divergence events during evolution. Additionally, cross-species comparison revealed that monocots generally have more CBL-CIPK genes, potentially related to their broader stress adaptability.

This study provides the first systematic characterization of the CBL-CIPK gene family in the ice plant, laying a foundation for in-depth exploration of its saline–alkaline tolerance molecular mechanisms. Future research could focus on the following directions: (1) Functional validation: Verify the specific functions of key genes (e.g., *McCBL1*, *McCIPK1*) under salt stress through gene knockout or overexpression experiments. (2) Protein interaction studies: Use yeast two-hybrid or co-immunoprecipitation techniques to investigate the formation of the CBL-CIPK complex and its downstream regulatory network. (3) Metabolic regulation analysis: Combine metabolomics to dissect how the CBL-CIPK signaling pathway affects the synthesis of osmo-protectants (e.g., proline, betaine).

## 5. Conclusions

This study identified 9 CBL and 18 CIPK genes in the ice plant, systematically analyzing their structural characteristics, expression patterns, and evolutionary relationships. The results indicate that some CBL and CIPK genes may participate in the saline–alkaline tolerance process of the ice plant through pathways involving hormone signaling, ion balance, and osmo-protection. These findings not only enrich research on the plant CBL-CIPK signaling pathway but also provide potential targets for improving crop salt tolerance using genetic engineering approaches.

## Figures and Tables

**Table 1:** Number of plant CBL-CIPK gene family members.

Classification	Species	CBL	CIPK	Photosynthetic Type
Algae	*Mesostigma viride*	2	1	C3
	*Volvox carteri*	1	0	C3
Ferns	*Selaginella moellendorffii*	4	5	C3
Mosses	*Physcomitrella patens*	5	8	C3
Gymnosperms	*Ginkgo biloba*	5	17	C3
	*Gnetum montanum*	7	8	C3
Basal angiosperms	*Amborella trichopoda*	7	12	C3
	*Nymphaea colorata*	9	15	C3
Monocotyledons	*Oryza sativa*	10	47	C3
	*Sorghum bicolor*	11	48	C4
	*Saccharum spontaneum*	14	54	C4
	*Saccharum officinarum*	74	7	C4
	*Zea mays*	25	53	C4
	*Ananas comosus*	8	21	CAM
	*Dendrobium catenatum*	17	29	Facultative CAM plants
Dicotyledons	*Arabidopsis thaliana*	10	24	C3
	*Brassica rapa*	18	41	C3
	*Brassica oleracea*	17	45	C3
	*Mesembryanthemum crystallinum*	7	18	Facultative CAM plants
	*Hylocereus undatus*	8	27	CAM
	*Spinacia oleracea*	6	13	C3
	*Vitis vinifera*	11	23	C3
	*Helianthus annuus*	14	28	C3
	*Aquilegia coerulea*	7	17	CAM

**Table 2:** Physicochemical properties and subcellular localization of ice plant CBL-CIPK gene family members.

Gene	Num of Amino Acids	Molecular Weight	Isoelectric Point	Instability Index	Aliphatic Index	Hydrophilicity Coefficient	Subcellular Localization
*McCBL1*	223	25,396.97	4.71	39.57	97.4	−0.18	nucl
*McCBL2*	219	25,112.75	4.76	32.49	93.01	−0.206	cyto
*McCBL3*	410	47,339.71	4.64	40.35	93.17	−0.262	cyto
*McCBL4*	213	24,471.69	4.81	41.34	84.18	−0.236	chlo
*McCBL5*	221	25,620.26	4.71	52.21	89.5	−0.342	cyto
*McCBL6*	219	25,434.09	5.24	29.47	86.3	−0.43	cyto
*McCBL7*	226	25,880.42	4.8	35.93	89.34	−0.261	cyto
*McCBL8*	252	28,688.76	4.76	37.43	98.33	−0.03	chlo
*McCBL9*	226	26,371.12	5	49.16	92.3	−0.25	cyto
*McCIPK1*	454	51,318.55	8.9	30.37	93.59	−0.341	cyto
*McCIPK2*	495	55,330.92	6.51	42.6	83.15	−0.331	chlo
*McCIPK3*	453	50,507.81	7.58	37.74	95.92	−0.264	cyto
*McCIPK4*	457	51,572.96	7.66	36.76	86.19	−0.365	chlo
*McCIPK5*	447	50,779.19	6.05	40.81	93.04	−0.259	plas
*McCIPK6*	457	51,285.25	9.14	38.82	88.51	−0.31	mito
*McCIPK7*	484	53,608.84	7.97	32.36	89.24	−0.216	chlo
*McCIPK8*	476	53,910.13	9.1	40.96	80.08	−0.405	chlo
*McCIPK9*	453	51,118.89	6.36	32.3	93.66	−0.267	cyto
*McCIPK10*	443	50,296.10	8.54	38.11	92.6	−0.171	chlo
*McCIPK11*	462	52,190.72	8.6	38.95	83.96	−0.393	plas
*McCIPK12*	441	50,028.82	7.94	40.58	85.49	−0.293	cyto
*McCIPK13*	460	50,734.46	9.16	43.44	88.11	−0.225	chlo
*McCIPK14*	457	51,885.06	9.14	27.89	82.71	−0.314	cyto
*McCIPK15*	365	41,340.50	6.48	33.67	84.38	−0.343	chlo
*McCIPK16*	463	53,024.01	8.64	36.53	86.35	−0.505	cyto
*McCIPK17*	476	53,892.50	8.95	35.41	86.62	−0.312	cyto
*McCIPK18*	480	54,329.23	8.52	36.82	82.83	−0.463	cyto

## Data Availability

The genomic data analyzed in this study were obtained from public databases of the National Center for Biotechnology Information (NCBI). As this study involved secondary data analysis, no new raw data were generated.

## References

[1] Sato R., Kondo Y., Agarie S. (2023). The first released available genome of the common ice plant (*Mesembryanthemum crystallinum* L.) extended the research region on salt tolerance, C_3_-CAM photosynthetic conversion, and halophilism. F1000Research.

[2] Shen S., Li N., Wang Y., Zhou R., Sun P., Lin H., Chen W., Yu T., Liu Z., Wang Z. (2022). High-quality ice plant reference genome analysis provides insights into genome evolution and allows exploration of genes involved in the transition from C3 to CAM pathways. Plant Biotechnol. J..

[3] Chiang C.P., Yim W.C., Sun Y.H., Ohnishi M., Mimura T., Cushman J.C., Yen H.E. (2016). Identification of Ice Plant (*Mesembryanthemum crystallinum* L.) MicroRNAs Using RNA-Seq and Their Putative Roles in High Salinity Responses in Seedlings. Front. Plant Sci..

[4] Gamage Kaushalya Madhavi B., Mun Choi G., Entaz Bahar M., Eun Moon B., Eun Kim N., Lee H.-W., Tae Kim H. (2022). Assessment of different salt concentrations on the growth and phytochemical change of the ice plants. J. King Saud Univ.-Sci..

[5] Lee D., Kim S.J., Choi Y.J., Rho Y.H., Kang T.S., Kim Y.G., Kang K.S. (2025). The Glucose-Lowering Effect of *Mesembryanthemum crystallinum* and D-Pinitol: Studies on Insulin Secretion in INS-1 Cells and the Reduction of Blood Glucose in Diabetic Rats. Nutrients.

[6] Kang Y.W., Joo N.M. (2023). Optimization of Nutrient-Rich Ice Plant (*Mesembryanthemum crystallinum* L.) Paste Fresh Noodle Pasta Using Response Surface Methodology. Foods.

[7] Kim H.L., Jung Y., Kim H.I., Sung N.Y., Kim M.J., Han I.J., Kim G., Nho E.Y., Park S.Y., Han Y. (2023). Antidiabetic Effect of Fermented *Mesembryanthemum crystallinum* L. in db/db Mice Involves Regulation of PI3K-Akt Pathway. Curr. Issues Mol. Biol..

[8] Hong H.T.K., Trang P.T.H., Ho T.-T., Dang J., Sato R., Yoshida K., Silaguntsuti P., Agarie S. (2024). Reproductive growth characteristics of *Mesembryanthemum crystallinum* L. in High-Salinity stress conditions. Sci. Hortic..

[9] Sene J.H.B., Faye E., Tine A.K. (2023). Curbing the Salinization of Arable Land and Agronomically Restoring Salt-affected Soils, a food security challenge: Assessment and prospects, the case of Senegal, West Africa. Mosc. Univ. Soil Sci. Bull..

[10] Tsukagoshi H., Suzuki T., Nishikawa K., Agarie S., Ishiguro S., Higashiyama T. (2015). RNA-seq analysis of the response of the halophyte, *Mesembryanthemum crystallinum* (ice plant) to high salinity. PLoS ONE.

[11] Park K.S., Kim S.K., Cho Y.-Y., Cha M.K., Jung D.H., Son J.E. (2016). A coupled model of photosynthesis and stomatal conductance for the ice plant (*Mesembryanthemum crystallinum* L.), a facultative CAM plant. Hortic. Environ. Biotechnol..

[12] Fan S., Yang S., Shi K., Yang L., An M., Wang F., Qi Y., Feng M., Wang M., Geng P. (2024). Genome-wide identification of the LRX gene family in Cucurbitaceae and expression analysis under salt and drought stress in cucumber. Veg. Res..

[13] Peng Y., Zhu H., Wang Y., Kang J., Hu L., Li L., Zhu K., Yan J., Bu X., Wang X. (2025). Revisiting the role of light signaling in plant responses to salt stress. Hortic. Res..

[14] Wang Y., Sun S., Feng X., Li N., Song X. (2024). Two lncRNAs of Chinese cabbage confer Arabidopsis with heat and drought tolerance. Veg. Res..

[15] Zhang X.X., Ren X.L., Qi X.T., Yang Z.M., Feng X.L., Zhang T., Wang H.J., Liang P., Jiang Q.Y., Yang W.J. (2022). Evolution of the CBL and CIPK gene families in Medicago: Genome-wide characterization, pervasive duplication, and expression pattern under salt and drought stress. BMC Plant Biol..

[16] Wang Q., Zhao K., Gong Y., Yang Y., Yue Y. (2022). Genome-Wide Identification and Functional Analysis of the Calcineurin B-like Protein and Calcineurin B-like Protein-Interacting Protein Kinase Gene Families in Chinese Cabbage (*Brassica rapa* ssp. *pekinensis*). Genes.

[17] Tang R.J., Wang C., Li K., Luan S. (2020). The CBL-CIPK Calcium Signaling Network: Unified Paradigm from 20 Years of Discoveries. Trends Plant Sci..

[18] Yang M., Zhou B., Song Z., Tan Z., Liu R., Luo Y., Guo Z., Lu S. (2024). A calmodulin-like protein PvCML9 negatively regulates salt tolerance. Plant Physiol. Biochem..

[19] Arthikala M.-K., Blanco L., Alvarado-Affantranger X., Márquez-Guzmán J., Lara M., Nanjareddy K. (2023). Identification of CBL and CIPK Gene Families and Functional Characterization of PvCIPK7 as an Essential Regulator of Root Nodule Development and Nitrogen Fixation in Phaseolus vulgaris. J. Plant Biol..

[20] Huang L., Li Z., Fu Q., Liang C., Liu Z., Liu Q., Pu G., Li J. (2021). Genome-Wide Identification of CBL-CIPK Gene Family in Honeysuckle (*Lonicera japonica* Thunb.) and Their Regulated Expression Under Salt Stress. Front. Genet..

[21] Xiaolin Z., Baoqiang W., Xian W., Xiaohong W. (2022). Identification of the CIPK-CBL family gene and functional characterization of CqCIPK14 gene under drought stress in quinoa. BMC Genom..

[22] Bihani S.C., Tarushi, Srivastava A.K. (2025). Decoding the calcium signal: Structural insights into CBL-CIPK pathway in plants. Biochim. Biophys. Acta (BBA)-Gen. Subj..

[23] Ma L., Ye J., Yang Y., Lin H., Yue L., Luo J., Long Y., Fu H., Liu X., Zhang Y. (2019). The SOS2-SCaBP8 Complex Generates and Fine-Tunes an AtANN4-Dependent Calcium Signature under Salt Stress. Dev. Cell.

[24] Ma X., Li Q.-H., Yu Y.-N., Qiao Y.-M., Haq S.U., Gong Z.-H. (2020). The CBL–CIPK Pathway in Plant Response to Stress Signals. Int. J. Mol. Sci..

[25] Mao J., Mo Z., Yuan G., Xiang H., Visser R.G.F., Bai Y., Liu H., Wang Q., van der Linden C.G. (2023). The CBL-CIPK network is involved in the physiological crosstalk between plant growth and stress adaptation. Plant Cell Environ..

[26] Huang Z., Chen S., He K., Yu T., Fu J., Gao S., Li H. (2024). Exploring salt tolerance mechanisms using machine learning for transcriptomic insights: Case study in Spartina alterniflora. Hortic. Res..

[27] Gharat S.A., Parmar S., Tambat S., Vasudevan M., Shaw B.P. (2016). Transcriptome Analysis of the Response to NaCl in Suaeda maritima Provides an Insight into Salt Tolerance Mechanisms in Halophytes. PLoS ONE.

[28] Chen C., Wu Y., Li J., Wang X., Zeng Z., Xu J., Liu Y., Feng J., Chen H., He Y. (2023). TBtools-II: A “one for all, all for one” bioinformatics platform for biological big-data mining. Mol. Plant.

[29] Wu T., Liu Z., Yu T., Zhou R., Yang Q., Cao R., Nie F., Ma X., Bai Y., Song X. (2024). Flowering genes identification, network analysis, and database construction for 837 plants. Hortic. Res..

[30] Feng S., Liu Z., Chen H., Li N., Yu T., Zhou R., Nie F., Guo D., Ma X., Song X. (2024). PHGD: An integrative and user-friendly database for plant hormone-related genes. iMeta.

[31] Mistry J., Chuguransky S., Williams L., Qureshi M., Salazar G.A., Sonnhammer E.L.L., Tosatto S.C.E., Paladin L., Raj S., Richardson L.J. (2021). Pfam: The protein families database in 2021. Nucleic Acids Res..

[32] Liu Z., Zhang C., He J., Li C., Fu Y., Zhou Y., Cao R., Liu H., Song X. (2024). plantGIR: A genomic database of plants. Hortic. Res..

[33] Shen L., Yang S., Xia X., Nie W., Yang X. (2024). Genome-wide identification of Kip-related protein (KRP) gene family members in eggplant and the function of *SmKRP3* under salt stress. Veg. Res..

[34] Emms D.M., Kelly S. (2019). OrthoFinder: Phylogenetic orthology inference for comparative genomics. Genome Biol..

[35] Katoh K., Misawa K., Kuma K., Miyata T. (2002). MAFFT: A novel method for rapid multiple sequence alignment based on fast Fourier transform. Nucleic Acids Res..

[36] Price M.N., Dehal P.S., Arkin A.P. (2010). FastTree 2—Approximately maximum-likelihood trees for large alignments. PLoS ONE.

[37] Letunic I., Bork P. (2024). Interactive Tree of Life (iTOL) v6: Recent updates to the phylogenetic tree display and annotation tool. Nucleic Acids Res..

[38] Tamura K., Stecher G., Kumar S. (2021). MEGA11: Molecular Evolutionary Genetics Analysis Version 11. Mol. Biol. Evol..

[39] Bailey T.L., Johnson J., Grant C.E., Noble W.S. (2015). The MEME Suite. Nucleic Acids Res..

[40] Lescot M., Dehais P., Thijs G., Marchal K., Moreau Y., Van de Peer Y., Rouze P., Rombauts S. (2002). PlantCARE, a database of plant cis-acting regulatory elements and a portal to tools for in silico analysis of promoter sequences. Nucleic Acids Res..

[41] Ono K., Fong D., Gao C., Churas C., Pillich R., Lenkiewicz J., Pratt D., Pico A.R., Hanspers K., Xin Y. (2025). Cytoscape Web: Bringing network biology to the browser. Nucleic Acids Res..

[42] Cushman J.C., Tillett R.L., Wood J.A., Branco J.M., Schlauch K.A. (2008). Large-scale mRNA expression profiling in the common ice plant, *Mesembryanthemum crystallinum*, performing C3 photosynthesis and Crassulacean acid metabolism (CAM). J. Exp. Bot..

[43] Chen J.S., Wang S.T., Mei Q., Sun T., Hu J.T., Xiao G.S., Chen H., Xuan Y.H. (2024). The role of CBL–CIPK signaling in plant responses to biotic and abiotic stresses. Plant Mol. Biol..

[44] Kaya C., Uğurlar F., Adamakis I.-D.S. (2024). Molecular Mechanisms of CBL-CIPK Signaling Pathway in Plant Abiotic Stress Tolerance and Hormone Crosstalk. Int. J. Mol. Sci..

